# The Use of Intraoperative Cholangiography During Cholecystectomy: A Systematic Review

**DOI:** 10.7759/cureus.47646

**Published:** 2023-10-25

**Authors:** Samah Osailan, Muhanad Esailan, Abdulaziz M Alraddadi, Faisal M Almutairi, Zaid Sayedalamin

**Affiliations:** 1 General Surgery, King Abdulaziz University Faculty of Medicine, Jeddah, SAU; 2 Medicine, Al-Rayan Colleges, Al-Madinah, SAU; 3 Medicine and Surgery, Al-Rayan Colleges, Al-Madinah, SAU

**Keywords:** intraoperative radiography, gallbladder surgery, choledocholithiasis, cholelithiasis, cholecystitis, laparoscopic cholecystectomy, cholecystectomy, intraoperative cholangiography

## Abstract

Cholecystectomy is a widespread surgical procedure for gallbladder diseases. Evolving techniques and technologies, such as intraoperative cholangiography (IOC), enhance safety and outcomes by providing real-time biliary system visualization during surgery. This systematic review explored available data on using IOC during cholecystectomy, highlighting its effectiveness, safety, and cost-effectiveness.

To perform this systematic review, a thorough literature search was conducted using relevant keywords in electronic databases, such as PubMed, Medical Literature Analysis and Retrieval System Online (MEDLINE), Cochrane Library, Web of Science, and Google Scholar. We included studies published during the last 10 years exploring the use of IOC during cholecystectomy.

The findings showed success rates of up to 90% with a median time of 21.9 minutes without complications. Most (90%) patients with acute gallstone pancreatitis underwent cholecystectomy with IOC, with unclear IOC results in 10.7% and failure in 14.7%. IOC failure factors included age, body mass index (BMI), male sex, concurrent acute cholecystitis, common bile duct (CBD) stone evidence on imaging, CBD diameter of >6 mm, total bilirubin of >4 mg/dL, abnormal liver tests, and gallstone pancreatitis. The detection of choledocholithiasis by IOC prompted trans-cystic duct exploration and endoscopic retrograde cholangiopancreatography (ERCP). Biliary abnormalities and stone identification were observed using IOC, and routine use increased bile duct stone detection while decreasing bile duct injury and readmission rates. The sensitivity, specificity, accuracy, positive predictive value, and negative predictive value of IOC for common bile duct stone detection were reported at 77%, 98%, 97.2%, 63%, and 99%, respectively. Routine IOC was projected to provide substantial quality-adjusted life years (QALY) and cost-effectiveness gains compared to selective IOC. Regarding safety, IOC was generally associated with reduced complication and open surgery conversion risks, with similar rates of CBD injury and bile leaks. These findings indicate that IOC enhances cholecystectomy outcomes through precision and decreasing complications.

## Introduction and background

Cholecystectomy is one of the most common and important interventions used to treat gallbladder pathologies [1]. Cholecystectomy has significantly changed over the years, with new techniques and technologies constantly improving patient results and safety. Incorporating intraoperative cholangiography (IOC) during cholecystectomy, allowing real-time viewing of the biliary system, is a critical innovation [2-4]. The biliary system, which transports and stores bile produced by the liver, is essential for digestion and metabolism [5]. The gallbladder, a small, pear-shaped organ on the right side of the abdomen beneath the liver, stores bile, a digestive fluid discharged into the small intestine for digestion [6]. Pathologies of the gallbladder, such as gallstones or inflammation, can cause serious consequences, necessitating the surgical removal of the gallbladder, known as cholecystectomy [1,7]. However, the biliary anatomy's complexity presents difficulties during this treatment, as an injury to the common bile duct (CBD) can have serious implications [8,9]. This is when IOC comes into play as a valuable method to minimize injury risks.

Intraoperative cholangiography involves the radiographic imaging of the biliary system during surgery [10,11]. During this procedure, a surgeon exposes the cystic duct and injects a contrast chemical into it. Fluoroscopic pictures are taken in real time as the contrast agent passes through the biliary system, allowing a dynamic visualization of the gallbladder, cystic duct, common bile duct, and any potential anomalies [7,10]. This real-time imaging helps the surgeon examine the anatomy, locate obstructions or stones, and protect sensitive structures. IOC is beneficial when the biliary anatomy is ambiguous or the surgeon meets unexpected challenges during surgical operations [12]. IOC provides crucial insights into the existence of stones or strictures inside the biliary system, assisting in surgical decision-making.

Furthermore, IOC safeguards against iatrogenic injury to the common bile duct, which might result in biloma, intraabdominal abscess and infection, and sepsis [4]. IOC helps to reduce postoperative complications by confirming the absence of residual stones and ensuring adequate biliary flow before the surgeons end the operation. It was found that the routine use of IOC in laparoscopic cholecystectomy (LC) was associated with a reduced risk of complications (odds ratio {OR}, 0.27; 95% confidence interval {CI}, 0.15-0.50; P < 0.001) and conversion to open cholecystectomy (OR, 0.11; 95% CI, 0.03-0.37; P < 0.001) [13].

While some studies suggest the benefits of intraoperative cholangiography (IOC) during cholecystectomy, its routine use remains a point of contention among surgeons [14,15]. Critics argue that IOC may increase surgical time, expose patients to more radiation, and lead to postoperative complications when compared to elective IOC (P = 0.04) [16]. Moreover, the likelihood of common bile duct injury after cholecystectomy is low in skilled hands, raising questions about the necessity of IOC in all cases. On the other hand, proponents contend that the potential benefits of IOC, especially in situations involving complex biliary anatomy, acute inflammation, or previous procedures, outweigh the perceived risks [11]. Despite these arguments, there is a clear lack of a comprehensive guide addressing when, how, and for whom IOC should be employed during cholecystectomy, considering the trade-offs between benefits and potential risks. This underscores the need for further investigation to provide evidence-based guidelines regarding the use of IOC in cholecystectomy procedures, taking into account patient outcomes, safety, and surgical efficiency, especially in specific clinical scenarios. Therefore, to address this gap, this systematic review aimed to explore the comparative effectiveness of routine IOC versus selective IOC to clarify its role and use in cholecystectomy.

## Review

Methods

The search question our systematic review aimed to answer was, "What is the impact of intraoperative cholangiography on outcomes in cholecystectomy procedures?"

Search Strategy

We conducted a systematic literature search of different electronic databases, including PubMed, Medical Literature Analysis and Retrieval System Online (MEDLINE), Cochrane Library, Web of Science, and Google Scholar, to identify relevant studies investigating the use of IOC during cholecystectomy. During searching, we used the following search terms: "Intraoperative cholangiography," "Intraoperative bile duct imaging," "IOC during cholecystectomy," "Cholecystectomy with cholangiography," "Common bile duct evaluation during surgery," and "Biliary imaging during surgery." We also used the relevant Medical Subject Headings (MeSH), such as "Cholangiography," "Cholecystectomy," "Gallbladder Diseases," "Gallbladder Surgery," "Bile Ducts," and "Bile Duct Injuries," and their combinations using Boolean operators (AND and OR) to optimize the search strategy. We manually searched the reference lists of relevant articles in addition to computerized database searches to uncover potential new studies that were missed during the original search.

Study Selection

**Figure 1:**
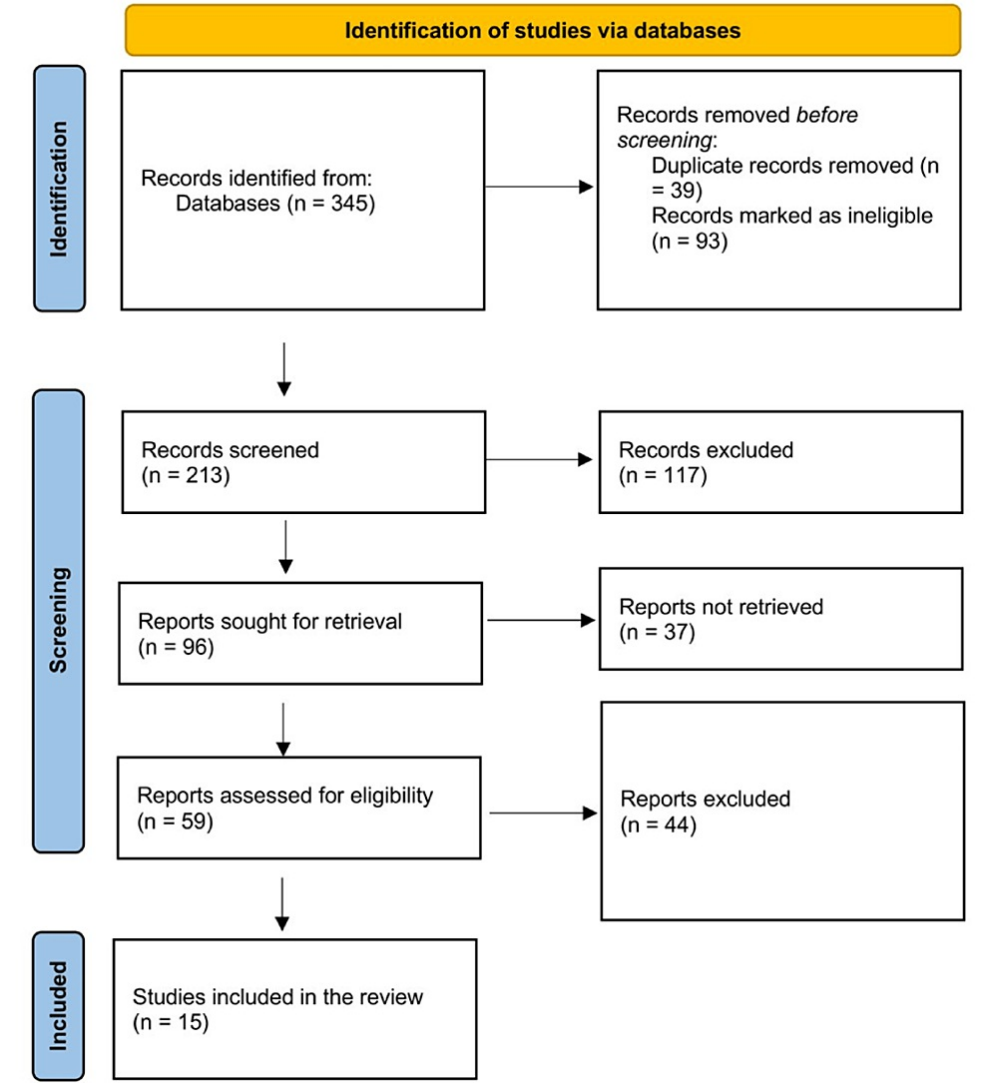
PRISMA flow diagram showing the selection process PRISMA: Preferred Reporting Items for Systematic Reviews and Meta-Analyses

Three reviewers selected studies based on the following criteria: randomized controlled trials (RCTs), cohort studies, case-control studies, and prospective/retrospective studies that involved patients undergoing cholecystectomy and reporting on the use of IOC during cholecystectomy. Studies that report clinical outcomes were included, and only studies published in English within the last 10 years were included to encounter the latest surgical and radiological technology advancements. We excluded duplicate studies, editorials, letters to the editor, opinion articles, narrative and scoping reviews, theses, and non-peer-reviewed articles. We retrieved and reviewed full-text publications of potentially relevant articles and assessed them for inclusion. Discrepancies among reviewers were settled by discussion, and if necessary, a fourth reviewer intervened. Figure 1 depicts the process of selecting the included studies.

Data Extraction and Quality Assessment

We used a standardized data extraction form to obtain data from the selected publications, noting first authors' names, year of publication, study design, and key relevant findings. Four reviewers extracted data independently, and any differences were resolved through discussion or consultation with a fifth reviewer, as needed. Four reviewers used appropriate tools based on study design to assess the quality of included studies, such as the Cochrane Risk of Bias tool for randomized controlled trials [17], the Newcastle-Ottawa Scale for observational studies [18], and the National Institutes of Health (NIH) Study Quality Assessment Tools for other studies [19]. The potential causes of bias in studies were examined, including selection, performance, detection, attrition, and reporting biases.

Data Synthesis and Reporting

We used a narrative synthesis approach to summarize extracted data and to offer an overview of the effectiveness, safety, and cost-effectiveness of using IOC during cholecystectomy. The findings were synthesized and reported based on the primary outcomes (common bile duct injuries, postoperative complications, and length of hospital stay) and secondary outcomes (cost-effectiveness and safety). In this systematic review, we followed the Preferred Reporting Items for Systematic Reviews and Meta-Analyses (PRISMA) guidelines for reporting. Due to a wide heterogeneity among included studies, a meta-analysis was not feasible.

Results

The initial search generated 345 article titles and abstracts, and after removing duplicates and other irrelevant titles and abstracts, 213 titles and abstracts were screened. The full-text versions of 96 articles were retrieved from relevant titles and abstracts, and 59 papers were assessed for eligibility. After thoroughly reviewing these full-text papers, 15 articles fulfilled all inclusion criteria (Table 1). Most of the included articles (eight) were retrospective studies, three were prospective studies, two were systematic reviews and meta-analyses, one was only a meta-analysis, and another used a Markov model decision analysis.

**Table 1:** Characteristics of the included studies CI, confidence interval; OR, odds ratio; $, US dollar; HR, hazard ratio

Authors	Year	Title	Study design	Summary of findings
Thacoor et al. [20]	2019	The role of intraoperative cholangiography in patients undergoing laparoscopic cholecystectomy for acute gallstone pancreatitis: is magnetic resonance cholangiopancreatography needed?	Retrospective study	Between October 1998 and December 2013, a total of 2,215 patients underwent laparoscopic cholecystectomy (LC). Ninety percent of patients with acute gallstone pancreatitis underwent laparoscopic cholecystectomy accompanied by intraoperative cholangiography (IOC). Intraoperative cholangiography revealed choledocholithiasis in 13 patients, of whom 11 received simultaneous treatment through trans-cystic duct exploration and clearance, and two patients necessitated postoperative endoscopic retrograde cholangiopancreatography (ERCP).
Reeves et al. [21]	2022	The price is right: Routine fluorescent cholangiography during laparoscopic cholecystectomy	Markov model decision analysis	The model's findings demonstrate that fluorescent cholangiography offers significant benefits over standard bright-light laparoscopic cholecystectomy, reducing lifetime costs by $1,235 per patient and enhancing effectiveness by 0.09 quality-adjusted life years (QALY) due to shorter operation durations (reduced by 21.20 minutes) and a lower open conversion rate (1.62% versus 6.70%). Probabilistic sensitivity analysis confirmed that in nearly 99% of model iterations, fluorescent cholangiography is more effective and less costly, even considering a willingness-to-pay threshold of $100,000 per quality-adjusted life year.
Esposito et al. [11]	2023	Systematic intraoperative cholangiography during elective laparoscopic cholecystectomy: Is it a justifiable practice?	Retrospective cohort study	Of 303 patients, 215 (71.0%) belonged to the IOC group, while 88 (29.0%) were in the non-IOC group. Incomplete or unclear IOC was found in 10.7% of cases, with a failure rate of 14.7%. The IOC group experienced a 15-minute longer operation time (P = 0.01) and exhibited higher postoperative complications (5.1% versus 0.0%, P = 0.03). All three cases of bile duct injuries (0.99%) were within the IOC group, one diagnosed intraoperatively and the other postoperatively. In terms of common bile duct (CBD) stone detection, IOC demonstrated 77% sensitivity, 98% specificity, 97.2% accuracy, a positive predictive value of 63%, and a negative predictive value of 99%.
Martin et al. [22]	2018	Selective intraoperative cholangiography during laparoscopic cholecystectomy in children is justified	Retrospective study	Intraoperative cholangiography found biliary abnormalities that required additional treatment in 6/62 (10%) of patients undergoing laparoscopic cholecystectomy. These findings support the use of intraoperative cholangiography in select individuals with CBD dilatation or preoperative imaging suspicion of ductal stones.
Rystedt et al. [23]	2021	Routine versus selective intraoperative cholangiography during cholecystectomy: systematic review, meta-analysis and health economic model analysis of iatrogenic bile duct injury	Systematic review and meta-analysis	Routine intraoperative cholangiography (IOC) detected 0.36% of bile duct injury, and selective IOC detected 0.53% of bile duct injury, significantly increasing the change of bile duct injury detection by selective IOC (OR, 1.43; 95% CI, 1.22-1.67). Through model analysis, it was projected that Sweden, with a population of 10 million, could avert seven injuries annually via routine IOC, consequently gaining 33 quality-adjusted life years (QALYs) over a decade. The associated net cost (€808,000) would amount to an approximate cost of €24,900 per QALY gained.
Lai et al. [24]	2022	Routine intraoperative cholangiography during laparoscopic cholecystectomy: application of the 2016 WSES guidelines for predicting choledocholithiasis	Retrospective study	A study included 990 patients who underwent laparoscopic cholecystectomy (LC) patients and routine intraoperative cholangiography (IOC). IOC revealed CBD stone in 19.9% of cases. Detection rates varied across low-, intermediate-, and high-risk groups. Predictors included the evidence of CBD stones on imaging, CBD diameter of >6 mm, total bilirubin of >4 mg/dL, abnormal liver tests, and clinical gallstone pancreatitis. The study identified major bile duct injuries in 0.4% of patients, all of whom successfully underwent repair surgery with uneventful recoveries.
Abdelaal et al. [2]	2017	Role of intraoperative cholangiography for detecting residual stones after biliary pancreatitis: still useful? A retrospective study	Retrospective study	In 84 out of 113 patients (74.3%), intraoperative cholangiography (IOC) revealed the presence of stones. A comparison between patients with and without stones found similar mean durations from hospital admission to surgery (5.9 days versus 6.1 days), from surgery to hospital discharge (2.0 days versus 2.2 days), and overall length of hospital stay (7.9 days versus 8.3 days) (P > 0.001).
Akingboye et al. [25]	2021	Outcomes From Routine Use of Intraoperative Cholangiogram in Laparoscopic Cholecystectomy: Factors Predicting Benefit From Selective Cholangiography	Systematic review and meta-analysis	Among 804 patients, 744 underwent intraoperative cholangiography (IOC). Filling defects were observed in 43 out of 744 patients (5.8%), with 23 out of the 43 cases undergoing stone extraction through endoscopic retrograde cholangiopancreatography (ERCP). Alkaline phosphatase (ALP) was a significant predictor of filling defects in IOC (OR, 1.003; 95% CI, 1.001-1.005; P = 0.015).
Ding et al. [12]	2015	Is intraoperative cholangiography necessary during laparoscopic cholecystectomy for cholelithiasis?	Prospective comparative study	The study included 371 participants aged 16-70, split into routine laparoscopic cholecystectomy (LC) (185) and LC + IOC (186) groups. Both groups were comparable in terms of demographics, gallstone attributes, and clinical symptoms. The rates of successful LC, CBD stone retainment, CBD injury, complications, and hospital stay duration showed no significant differences between groups. However, the LC + IOC group experienced a significantly longer mean operative time (52.86 ± 4.47 minutes versus 43.00 ± 4.15 minutes, P < 0.01). No fatal complications emerged, and a one-year follow-up identified minor digestive discomfort without abnormal radiological findings.
Tomaoğlu [26]	2020	Intraoperative Cholangiography in Laparoscopic Cholecystectomy: Technique and Changing Indications	Retrospective study	Of the 29 patients, 20 were females, and nine were males, with a mean age of 54.4 years. Successful IOC was achieved in 90% of cases, with a median duration of 21.9 minutes. Anatomical aberration was observed in one patient, wherein the cystic duct was connected to the right hepatic duct. The visualization of the Wirsung duct in another patient was due to the sphincter of Oddi hypertension. The procedure itself did not lead to any complications.
Silva et al. [27]	2013	Intraoperative cholangiography during elective laparoscopic cholecystectomy: selective or routine use?	Prospective study	Among the 243 patients, 33 (13.58%) were identified with choledocholithiasis. Of the 100 patients without an initial indication for this examination, only one case (1.0%) unveiled previously undetected choledocholithiasis. However, among the 143 patients with a preoperative indication for IOC, 32 (22.37%) cases of choledocholithiasis were observed.
Johansson et al. [28]	2021	Intervention versus surveillance in patients with common bile duct stones detected by intraoperative cholangiography: a population-based registry study	Retrospective study	The study included 134,419 patients who underwent cholecystectomy, with 2.0% undergoing ERCP for retained CBD stones. After accounting for factors such as cholecystectomy type, preoperative symptoms, age, and gender, the absence of IOC increased ERCP risk (HR, 1.4; 95% CI, 1.3-1.6). When CBD stones identified via IOC were managed through surveillance, the ERCP risk increased (HR, 5.5; 95% CI, 4.8-6.4). Even asymptomatic small stones (<4 mm) in the surveillance group had elevated ERCP risk compared to the intervention group (HR, 3.5; 95% CI, 2.4-5.1).
Iranmanesh et al. [29]	2018	Feasibility, benefit and risk of systematic intraoperative cholangiogram in patients undergoing emergency cholecystectomy	Retrospective study	Successful IOC was achieved in 509 out of 578 patients (88.1%). Primary factors influencing IOC failure were age, body mass index, male sex, and concurrent acute cholecystitis. Among patients with anticipated common bile duct stones during IOC, 32 underwent unnecessary negative postoperative assessments (6.3% of 509). A single adverse event related to IOC was recorded (mild pancreatitis, 0.2% of 578).
Askari et al. [13]	2021	Benefits of intraoperative cholangiogram for acute cholecystitis	Prospective study	Most (84.6%) patients underwent IOC. The overall complication rate was 8.1% (n = 55/676), notably lower in the IOC group (6.1%) compared to the non-IOC group (19.2%, P < 0.001). Specifically, there were reduced rates of retained stones (1.6% versus 3.8%, P < 0.001), bleeding (0.0% versus 2.9%, P < 0.001), and conversion to open surgery (0.7% versus 7.7%, P < 0.001). CBD injury rates (0.0% versus 0.3%, P = 0.5465) and bile leaks were comparable across groups (1.9% versus 0.9%). There was an association between IOC usage and lowered complication risk (OR, 0.27; 95% CI, 0.15-0.50; P < 0.001) and reduced conversion to open surgery (OR, 0.11; 95% CI, 0.03-0.37; P < 0.001).
Donnellan et al. [30]	2021	A meta-analysis of the use of intraoperative cholangiography; time to revisit our approach to cholecystectomy?	Meta-analysis	Routine intraoperative cholangiography led to the increased detection of bile duct stones during cholecystectomy compared to selective intraoperative cholangiography (OR, 3.28; 95% CI, 2.80-3.86; P < 0.001). Although bile duct injury incidence was slightly lower with intraoperative cholangiography (0.39%) than without (0.43%), the difference was not statistically significant (OR, 0.88; 95% CI, 0.65-1.19; P = 0.41). Readmission rates post cholecystectomy with intraoperative cholangiography were 3.0% and 3.5% without it (OR, 0.91; 95% CI, 0.78-1.06; P = 0.23).

The included studies reported different types of cholecystectomies (both open and laparoscopic) and IOC methods [11,20,24-26], with up to 90% success rate, median time of 21.9 minutes, and without complications [26]. One study reported that between 1998 and 2013, 2,215 patients had laparoscopic cholecystectomy (LC), and 90% of patients with acute gallstone pancreatitis had cholecystectomy and intraoperative cholangiography [20]. A cohort reported that unclear IOC was in 10.7%, and the other 14.7% failed [11]. Moreover, filling defects were in 5.8%, and high alkaline phosphatase (ALP) predicted filling defects (OR, 1.003; 95% CI, 1.001-1.005; P = 0.015) in IOC [25]. Demographic factors influencing IOC failure include age, body mass index (BMI), male sex, and concurrent acute cholecystitis [29]. Furthermore, other reported predictors included CBD stone evidence on imaging, CBD diameter of >6 mm, total bilirubin of >4 mg/dL, abnormal liver tests, and gallstone pancreatitis [24].

Different benefits and the effectiveness of IOC were reported by 11 included studies [2,11,12,20-24,27,28,30]. Choledocholithiasis was observed through intraoperative cholangiography, leading to trans-cystic duct exploration and endoscopic retrograde cholangiopancreatography (ERCP) [20]. A study comparing fluorescent cholangiography to standard bright-light laparoscopic cholecystectomy found notable benefits of fluorescent cholangiography: cost savings of $1,235 per patient, 0.09 quality-adjusted life years gained, 21.20-minute shorter operations, and lower open conversion rate (1.62% versus 6.70%) [21]. Another study showed that IOC identified stones in 74.3%, with no significant differences in admission to surgery, surgery to discharge, or overall hospital stay durations [2]. It was found that IOC identified biliary abnormalities in laparoscopic cholecystectomy patients, CBD dilatation or suspected ductal stones, and CBD stones, varying across risk groups [22-24]. The comparison of routine laparoscopic cholecystectomy (LC) and LC + IOC showed similar outcomes, except for a longer operative time in LC + IOC (52.86 versus 43.00 minutes) [12]. Among 243 patients scheduled for elective laparoscopic cholecystectomy, routine IOC detected 13.58% choledocholithiasis, rising to 22.37% for those with preoperative IOC indication [27]. Another study of 134,419 cholecystectomy patients discovered higher ERCP risk without IOC (hazard ratio {HR}, 1.4; 95% CI, 1.3-1.6) and increased risk with the surveillance of identified CBD stones (HR, 5.5; 95% CI, 4.8-6.4) [28]. The comparison of routine IOC to selective IOC found that routine IOC increased bile duct stone detection with lower bile duct injury incidence and readmission rate [23,30]. Regarding common bile duct stone detection, one study reported that IOC demonstrated 77% sensitivity, 98% specificity, 97.2% accuracy, a positive predictive value of 63%, and a negative predictive value of 99% [11]. A Swedish analysis showed that routine IOC could help gain 33 quality-adjusted life years (QALY) over a decade at an approximate cost of €24,900 per QALY gained (€808,000 net cost) [23]. Consistent with this report, intraoperative fluorescent cholangiography was reported to be more effective and less costly, even considering a willingness-to-pay threshold of $100,000 per QALY [21].

Regarding safety, there was an association between IOC usage and the lower risk of complications (OR, 0.27; 95% CI, 0.15-0.50; P < 0.001) and reduced conversion to open surgery (OR, 0.11; 95% CI, 0.03-0.37; P < 0.001), with similar CBD injury rates (0.0% versus 0.3%) and bile leaks (1.9% versus 0.9%) between patients undergoing IOC and those without IOC during cholecystectomy [13]. However, one study found that routine IOC during elective laparoscopic cholecystectomy was associated with longer operation times (15 minutes) and higher postoperative complications (5.1% versus 0.0%, P = 0.03), including bile duct injuries [11].

Discussion

This systematic review aimed to explore the comparative effectiveness of routine IOC versus selective IOC to clarify its role in modern surgical practice. This study would provide evidence-based insights into the role and effectiveness of IOC during cholecystectomy, guiding surgeons and ultimately leading to improved clinical practice, patient safety, and efficiency in surgical procedures.

The findings showed that IOC was effective in enhancing anatomical visualization during cholecystectomy, was safe, and reduced complications while leading to positive outcomes. Cholecystectomy is a frequent technique used to treat various gallbladder-related problems, mainly gallstones and inflammation [31,32]. Open cholecystectomy is an invasive procedure associated with the risk of injury, especially bile duct injury, due to limited operating intraabdominal space with tightly spaced structures [1]. Though laparoscopic cholecystectomy is a minimally invasive procedure that reduces postoperative pain, hospital stay, and recovery time, it is also challenging due to the lack of tactile feedback and limited visibility for the surgeon [27,33]. IOC offers real-time imaging of the biliary system to improve surgical precision and patient safety during cholecystectomy [12,34]. IOC involves injecting contrast material into the biliary tract and then imaging with fluoroscopy or radiography, enabling surgeons to see the structure of the bile ducts and identify any abnormalities, such as common bile duct stones or structural variations, that would otherwise be missed by direct inspection [35].

The findings showed that IOC enhances CBD exploration. If stones are not discovered and handled properly, they can move from the gallbladder into the CBD (choledocholithiasis), causing postoperative complications, such as cholangitis or gallstone pancreatitis, that lead to further surgical procedures such as endoscopic retrograde cholangiopancreatography (ERCP) or open surgery. IOC results guide trans-cystic duct exploration and endoscopic retrograde cholangiopancreatography to address choledocholithiasis [35,36]. Studies indicated that IOC is safe, enabling real-time visualization procedures to explore bile duct anatomy during laparoscopic cholecystectomy [37]. Biliary anatomy has a wide variation, and IOC assists surgeons in identifying anatomical defects such as ductal branching patterns or auxiliary ducts [38], enabling them to avoid iatrogenic injuries to vital structures during surgery. Studies indicated that by helping to identify and address biliary stones in consideration of anatomical variations, IOC reduces postoperative complications, such as retained stones, bile leaks, and CBD injuries or surrounding vital structures [16,39-41]. These studies align with our findings that showed lower bleeding rates, bile leaks, postoperative complications, retained stones, and conversion to open surgery.

Another previous systematic review and meta-analysis found that IOC was linked to a reduced risk of conversion to open surgery (risk ratio {RR}, 0.64; 95% confidence interval {CI}, 0.51-0.78) [42]. Moreover, IOC gives real-time visual input to surgeons, increasing their confidence and decision-making during the process [43]. This is particularly useful during laparoscopic cholecystectomy, where tactile feedback may be limited [12,34]. Our findings showed that IOC has 77% sensitivity, 98% specificity, 97.2% accuracy, and a positive predictive value of 63% in identifying CBD stones. These align with 10.7% unclarity and 14.7% failure, also shown by our findings. These numbers show that IOC is a safer and more accurate method compared to ultrasound sensitivity of 60% and specificity of 77% in detecting bile duct stones [44,45]. One previous study showed a slightly better sensitivity of 100% but a lower specificity of 53.1% and an accuracy of 57.1% in detecting the conversion to open surgery [43]. Several factors, such as male gender, old age, BMI, a history of abdominal surgery, acute cholecystitis with fever, leukocytosis, the presence of gallbladder stones, and certain ultrasonographic findings (the distension of the gallbladder, thick gallbladder lining, and impacted stone) predict the difficulties during IOC [46]. These factors are similar to factors found in our systematic review and highlight the need for surgeons to consider them when performing and interpreting IOC.

Our findings showed that the use of routine IOC was cost-effective compared to cholecystectomy without IOC, and it also led to QALY gain, by reducing postoperative complications, hospital stay, the duration of operation, and pain that were found to be associated with increased spending and resource consumption [47,48]. It was found that postoperative complications increased hospital costs by 78% and the length of hospital stay by up to 114% [49]. Therefore, IOC contributes to reducing surgery costs by improving care quality and outcomes [16]. These findings confirm that routine IOC is safe and effective, as previous studies suggest it should be required, particularly during laparoscopic cholecystectomy [29,34,50]. A previous systematic review and meta-analysis contrasted these findings by showing that routine IOC did not demonstrate superiority over selective IOC in reducing bile duct injuries (risk ratio {RR}, 0.91; 95% CI, 0.66-1.24; P = 0.805) since no statistically significant differences were observed for bile duct injuries, retained stone occurrence, readmission rates, and the length of hospital stay in comparing IOC group to non-IOC group. Furthermore, the IOC group exhibited significantly longer operation times in comparison to the group without IOC (weight mean difference {WMD}, 11.25 minutes; 95% CI, 6.57-15.93) [42]. Similarly, only one study in our systematic review found that routine IOC during elective laparoscopic cholecystectomy was associated with longer operation times (15 minutes) and higher postoperative complications (5.1% versus. 0.0%, P = 0.03). These indicate that the use of routine IOC remains controversial and needs further studies to clarify the debates surrounding routine IOC versus selective IOC. IOC might not be indicated for every patient undergoing cholecystectomy since the available evidence is ambiguous [16,42]. In addition to these benefits, IOC could be an educational tool, allowing surgeons in training to understand complex biliary anatomy and the interpretation of radiographic images.

There are some challenges associated with IOC. IOC may lengthen the surgical procedure, which may result in increased operating room time and associated costs [51], which might be explained by our finding that IOC was associated with 15 minutes of extra time. Patients and surgical staff are exposed to ionizing radiation with fluoroscopy or radiography during IOC. Though low and considered safe, avoiding unnecessary exposure is critical. The interpretation of IOC photographs necessitates extra skills and knowledge to accurately interpret radiography findings. Moreover, there are some indications to be considered for IOC to minimize unnecessary operations. These include the presence of pancreatitis, jaundice, elevated bilirubin levels, abnormal liver function test findings including increased liver enzymes, or a dilated CBD on preoperative ultrasonography.

## Conclusions

This systematic review showed that routine IOC has varying benefits, such as choledocholithiasis detection and bile duct variation visualization, leading to further exploration, reduced postoperative complications, and improved outcomes. IOC reduces open surgery conversion risks during laparoscopic cholecystectomy. These findings indicate that IOC is a safe and effective method for enhancing cholecystectomy outcomes. Its ability to increase surgical precision and reduce complications was highlighted. However, it should be used cautiously, considering clinical indications, such as dilated CBD, elevated liver enzymes, pancreatitis, and jaundice. Patient-specific circumstances and the surgeon's skill level should also be taken into consideration. As technology advances, incorporating procedures such as IOC illustrates the medical field's commitment to improving surgical practices and prioritizing patient safety.
